# A robust and scalable immunoadsorbent platform based on the high-affinity M03-IgG antibody for targeted IL-6 Removal in cytokine storm

**DOI:** 10.3389/fimmu.2026.1907563

**Published:** 2026-07-24

**Authors:** Yaxi Yang, Lei Wan, Youjia Qin, Jiangtao Guo, Yong Wang, Kangkang Ji, Sheng Hu, Mingqian Feng

**Affiliations:** 1 College of Biomedicine and Health, Huazhong Agricultural University, Wuhan, Hubei, China; 2 College of Life Science and Technology, Huazhong Agricultural University, Wuhan, Hubei, China; 3 Department of Respiratory and Critical Care Medicine, Binhai County People’s Hospital, Binhai Clinical College, Yangzhou University Medical College, Yancheng, Jiangsu, China; 4 Department of Oncology, Hubei Cancer Hospital, Tongji Medical College, Huazhong University of Science and Technology, Wuhan, Hubei, China

**Keywords:** cytokine removal, hypercytokinemia, il-6, immunoadsorption, inflammation

## Abstract

The therapeutic removal of interleukin-6 (IL-6) via blood perfusion has demonstrated clinical efficacy in the management of autoimmune diseases and sepsis. However, efficient clearance is hindered by the complexity of blood, which contains over 3,000 plasma proteins, and by the low physiological abundance of IL-6, which ranges from picograms to nanograms per milliliter even during cytokine storms. Existing adsorbents are limited by their specificity, binding capacity, and cost-effectiveness. To address these challenges, we developed an IgG-based immunoadsorbent utilizing M03-IgG, a high-affinity anti-IL-6 antibody with an EC_50_ of approximately 15 pM. Langmuir isotherm analysis indicated a maximum adsorption capacity of 5.18 mg of IL-6 per gram of microspheres. Dynamic adsorption assays, conducted at an adsorbent-to-serum ratio of 1:25, achieved approximately 93% clearance of IL-6. The adsorbent exhibited excellent blood compatibility and retained 85% of its initial activity following six months of storage. These results underscore the potential of this system as a clinically viable, high-performance platform for IL-6 removal.

## Graphical Abstract

**Figure:**
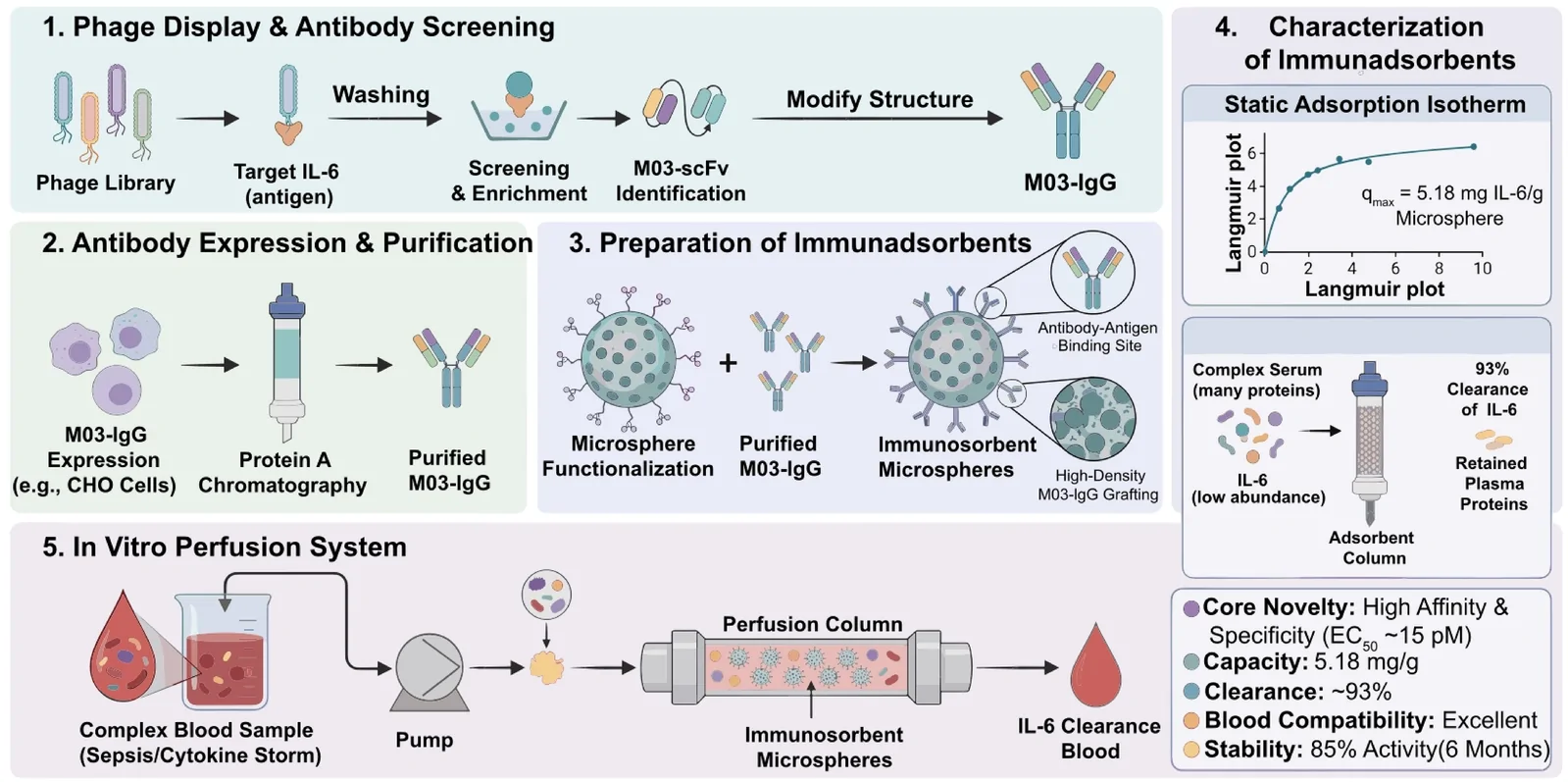
High-performance immunoadsorbent platform for targeted IL-6 clearance.

## Introduction

Inflammatory cytokines, a class of low−molecular−weight proteins that includes interleukins (IL), interferons (IFN), tumor necrosis factors (TNF), granulocyte-macrophage colony stimulating factor (GM-CSF), and chemokines, function as critical modulators of immune responses (1–4). Dysregulated cytokine release underlies both chronic autoimmune disorders, such as rheumatoid arthritis, psoriasis, and systemic lupus erythematosus, and acute conditions like sepsis and COVID−19 associated cytokine storms (CS) (5–8). Among these key pathogenic mediators, IL−6, IL−1β, TNF−α, and IFN−γ play pivotal roles (9–12).

IL-6 is a multifunctional cytokine that displays both pro-inflammatory and anti-inflammatory properties, a duality primarily arising from its two distinct signaling pathways (13–17). When IL-6 binds to membrane-bound IL-6 receptor (mIL-6R), it initiates the classical signaling pathway. This pathway involves gp130 homodimerization and primarily activates hepatocytes and specific immune cells, leading to the synthesis of acute-phase proteins, differentiation of regulatory T cells, and promotion of tissue repair. Thus, the classical pathway is generally regarded as anti-inflammatory and homeostatic in function (18). Conversely, IL-6 binds to soluble IL-6 receptor (sIL-6R), which is generated via proteolytic cleavage (primarily by ADAM17) or alternative splicing. The IL-6/sIL-6R complex then activates any cell expressing gp130 (e.g., endothelial, epithelial, and fibroblast cells) in a process termed trans-signaling, which is the main pathway driving chronic inflammation and tissue damage (19).

As an upstream regulator of the JAK/STAT pathway, IL−6 amplifies inflammation by inducing the expression of downstream factors such as IL−1β, TNF−α, IFN−γ, and IL−17 (20–23). Clinically, elevated IL−6 levels correlate with disease severity. For example, levels in patients with acute respiratory distress syndrome (ARDS) exceed 1000 pg/mL, which starkly contrasts with concentrations in healthy individuals (1–5 pg/mL) (24, 25). Clinically, IL−6 is neutralized by antibodies (tocilizumab/siltuximab) or soluble receptor constructs (26–28). Hemoadsorption is preferred when rapid removal is required, when neutralizing antibodies fail due to excessive antigen burden, or when multi−cytokine clearance is desired (e.g., septic shock, CAR−T therapy). Consequently, IL−6 serves as both a biomarker for CS−related severity and a therapeutic target. This dual role drives the demand for efficient IL−6 removal strategies (29–32).

Hemoadsorption (HA) is an extracorporeal technique that employs porous microsphere−based devices to remove inflammatory mediators. CytoSorb^®^, a representative resin−based adsorbent, leverages size−exclusion and hydrophobic interactions to capture molecules within the 6–70 kDa range, including cytokines, bilirubin, and myoglobin (33–36). For example, multiple studies have shown that in specific sepsis patient subgroups, CytoSorb^®^ treatment is associated with a reduction in serum cytokine levels (37, 38). However, its clinical efficacy remains inconsistent. In a study of 100 septic patients, CytoSorb^®^ therapy resulted in no reduction in IL−6 levels or mortality (39). Another study reported transient reductions in IL−6 (28%) and TNF−α (8.5%), followed by a rebound above baseline within four hours (40). These findings indicate inherent limitations in the adsorbent’s binding capacity and kinetics.

Immunoadsorption (IA) addresses this specificity gap by exploiting antibody−antigen recognition. Immunoglobulin G (IgG) antibodies, which are tetrameric proteins composed of variable (VH/VL) and constant domains, serve as ideal ligands because their antigen−binding fragment enables high−affinity capture (41–44).

Based on this principle, we hypothesized that immobilization of a phage-derived anti-IL-6 antibody onto epoxy-modified microspheres would produce a high-performance immunoadsorbent. In this study, we describe the development and characterization of this system, termed the M03 adsorbent. We also established a continuous-flow system to evaluate the performance of the M03-based immunoadsorbent (M03-adsorbent). The system was shown to exhibit IL-6 binding capacity, efficient removal performance, biocompatibility, and stability.

## Materials and methods

### Materials

The epoxy-functionalized microspheres LX1000EP, ES103B, N-ES108, and N-ES109 are all made of polymethyl methacrylate (PMMA). Healthy−donor plasma was prepared from citrate−anticoagulated whole blood provided by the Wuhan Blood Center (Wuhan, China). Phosphate−buffered saline (PBS; 10 mM phosphate, 150 mM NaCl, pH 7.4) and a bicinchoninic acid (BCA) protein quantification kit was acquired from Beijing Solarbio Science & Technology Co., Ltd. (Beijing, China). Glass chromatography columns (26 mm inner diameter × 100 mm length) were purchased from Beijing Jinding Biological Technology Co., Ltd. (Beijing, China). IL-6, IL-1β, TNF-α ELISA kits were obtained from Youke Life Co., Ltd. All other chemical reagents were of analytical grade. The therapeutic antibody siltuximab (for injection) was purchased from BeiGene Biopharma Co., Ltd. (Beijing, China).

### Antibody Selection via phage display

The recombinant human IL−6 (NP_000591.1), IL-1β (NP_000567.1) and TNF-α (NP_000585.2) gene were expressed in HEK 293F cells to produce two protein constructs: one with a C−terminal hexahistidine tag (IL−6−His, IL-1β−His, TNF-α−His) and another as a human Fc−fusion protein (IL−6−hFc, IL-1β−hFc, TNF-α−hFc). BALB/c mice were immunized with the IL−6−hFc protein. Following immunization, total RNA was extracted from splenocytes and used to construct a phage−display antibody library according to established protocols (45). Subsequently, four rounds of biopanning were performed against the IL−6−His antigen. This screening process yielded five distinct anti−IL−6 monoclonal antibodies, designated M03, M07, M19, M30, and M88.

### mAb production and purification

Selected single−chain variable fragment (scFv) constructs were genetically fused to a human immunoglobulin G1 Fc (IgG1−Fc) domain and cloned into the pFUSE mammalian expression vector. The variable heavy (VH) and variable light (VL) domains from the M03−scFv gene were subcloned into pFUSE−hIgG1 vectors, respectively, to produce the full−length human IgG1/kappa antibody. No substitutions were made in the CDR regions. Following transfection, the resulting fusion proteins were expressed via secretory pathways in Chinese hamster ovary (CHO) cells (Invitrogen) and subsequently purified using Protein A affinity chromatography. Purity was confirmed to exceed 90%, and molecular weight was verified by sodium dodecyl sulfate−polyacrylamide gel electrophoresis (SDS−PAGE). The purified antibodies were aliquoted and stored in phosphate−buffered saline (PBS; pH 7.4) at –80 °C.

### Affinity measurement by ELISA

IL-6-His, IL-1β−His, and TNF-α−His (5 µg/mL) was coated onto microtiter plates overnight at 4 °C. Serially diluted test antibodies were then added to the wells, and antibody binding was detected using a horseradish peroxidase (HRP)-conjugated goat anti-human IgG secondary antibody at a dilution of 1:5,000 (Biodragon Biotech). Half-maximal effective concentration (EC_50_) values were calculated from the resulting binding curves using non-linear regression in GraphPad Prism software (version 5).

### Immunosorbent preparation and characterization

*Coupling Procedure:* Epoxy-functionalized microspheres were incubated with antibody (1 mg/mL) or bovine serum albumin (BSA, 50 mg/mL) in PBS at a solution-to-microsphere volume ratio of 20:1 for 16 h at room temperature. After washing with PBS, the remaining epoxy groups were blocked with 6% ethanolamine (pH 10.0) for 6 h. The resulting immunomicrospheres were stored in PBS containing 0.02% NaN_3_ at 4 °C. *Key Parameters:* Coupling density: D_C_ (mg/g) = (C_0_ − C_1_) × V_N_/m (Eq. 1); Coupling efficiency: E_C_ = (C_0_ − C_1_) C_0_ ×100% (Eq. 2), where C_0_/C_1_ = initial/residual Ab concentration, V_N_ = volume, m = bead mass. *Dynamic Adsorption:* Columns (7.26 mm diameter × 56 mm length) packed with 0.5 g immunosorbent were perfused with IL-6 solution (20 ng/mL). Capacity and clearance were calculated as: C_A_ = (C_i_ − C_r_) × V/m (Eq. 3); R_C_ = (C_i_ − C_r_) C_i_ ×100% (Eq. 4), where Ci/Cr = initial/residual IL-6, V = volume, m = sorbent mass.

### Static adsorption analysis

Immunosorbents (0.5 g) were rotated with IL-6 (0.5 mg/mL in PBS, 4 mL) at RT. Kinetics were assessed by BCA measurement of supernatants at 0–60 min. For isotherms, beads were incubated with IL-6 (0-1500 μg/mL in serum, 4 mL, 37 °C, 30 min). Data were fitted to Langmuir models: q = (q_m_ × c_e_)/(K_d_ + c_e_) (Eq. 5); c_e_=q_m_ × c_e_/q - K_d_ (Eq. 6); q = (c_0_ − c_e_) × v_s_/m_g_ (Eq. 7), where q = adsorbed IL-6 (mg/g), q_m_= max capacity, K_d_ = dissociation constant.

### *In vitro* perfusion system

A simplified continuous-flow apparatus was constructed to simulate the clinical hemoperfusion process (Scheme 1). Twenty grams of immunosorbent microspheres or 20 g of unloaded microspheres (control) were packed into separate chromatographic columns (inner diameter, 26 mm; length, 100 mm) to ensure complete coverage of the sieve plates. Recombinant IL-6-His was added to 500 mL of thawed adult plasma. Perfusion was then performed under fixed conditions (adsorbent-to-plasma ratio, 1:25; flow rate, 30 mL/min; initial IL-6 concentration, 1600 pg/mL) to determine the adsorption equilibrium time. In subsequent experiments, the plasma volume (500 mL) and perfusion time (100 min) were kept constant, while the following variables were systematically varied: (1) flow rate (20–50 mL/min) at a fixed adsorbent-to-plasma ratio of 1:25 and an initial IL-6 concentration of 4500 pg/mL; (2) adsorbent-to-plasma ratio (1:25–1:100) at a fixed flow rate of 30 mL/min and an initial IL-6 concentration of 1400 pg/mL; and (3) initial IL-6 concentration (500–3000 pg/mL) at a fixed adsorbent-to-plasma ratio of 1:25 and a flow rate of 30 mL/min. The clearance rate was calculated as follows: Clearance rate (%) = [(C_0_ − C)/C_0_] × 100 (Eq. 8), where C_0_ and C represent the initial and steady-state concentrations of IL-6 in circulating plasma (pg/mL), respectively.

### Hemocompatibility assessment

Blood collected from healthy donors is centrifuged at 1,500 ×g for 20 min to obtain platelet-poor plasma. For protein adsorption and coagulation assessments, 20 mg of adsorbent is incubated with 1 mL of fresh plasma at 37°C for 60 min. The resulting supernatant was analyzed for plasma proteins, enzymes, metabolites, and bilirubin components, including fibrinogen (FIB), immunoglobulins (IgG, IgA, and IgM), alkaline phosphatase (ALP), alanine aminotransferase (ALT), aspartate aminotransferase (AST), γ−glutamyl transferase (γ−GT), direct bilirubin (BILD), total bilirubin (BILT), indirect bilirubin (BILI), urea (URE), glucose (Glu), and human complement fragments C3 and C4. Coagulation parameters, including prothrombin time (PT), activated partial thromboplastin time (APTT), and thrombin time (TT), are measured at Tongji Hospital. Hemolysis testing is conducted according to ASTM F756-2008 (46). Citrated whole blood (5 mL) is diluted 1:2 with Ca2+/Mg2+ -free saline and centrifuged at 2,000 rpm (≈ 400 ×g) for 10 min. The erythrocytes are resuspended in saline. Microspheres (20 mg) preincubated at 37°C for 30 min are mixed with 1 mL of red blood cell (RBC) suspension and incubated at 37°C for 3 h with continuous agitation. After centrifugation at 6,500 rpm (≈ 3,800 ×g) for 3 min, the hemoglobin released into the supernatant is quantified by measuring absorbance at OD540. Experimental controls consist of a negative control containing 0.2 mL RBC suspension plus 0.8 mL saline, and a positive control containing 0.2 mL RBC suspension plus 0.8 mL deionized water.

### Evaluation of antibody leaching and storage stability in immunoadsorbents

Recombinant IL-6-His (5 μg/mL, 50 μL/well) was coated onto a 96-well microplate and incubated overnight at 4 °C. After washing with PBST and blocking, add M03-IgG standards diluted in a 1:3 gradient (0.01–3000 ng/mL) and the tested outflow samples were added and incubated for 1h at room temperature. After washing, HRP-labeled goat anti-human IgG-Fc secondary antibody (1:5,000 dilution) was added and incubated for 1 hour at room temperature. After color development with TMB substrate, the absorbance was read at 450 nm. The concentration of M03-IgG in the samples was calculated based on the standard curve, and the lower limit of detection was determined to be 0.1 ng/mL. All samples were tested in triplicate.

Immunosorbents were stored in PBS (pH 7.4) at 4 °C. IL-6 adsorption capacity was evaluated monthly over 6 months.

### Statistical analysis

All statistical analyses were conducted using GraphPad Prism5(GraphPad Software, Inc., La Jolla, CA) and Origin Pro 2024 (Origin Lab, Northampton, MA). For one-way ANOVA and two-way ANOVA, if statistical significance is reached (p < 0.05), Tukey’s honestly significant difference test (for all pairwise comparisons among groups) or Dunnett’s test (for comparisons between all groups and the same control group) is further used for *post-hoc* multiple comparison correction.

## Results

### Preparation and functional validation of IL-6 monoclonal antibodies

**Figure 1:**
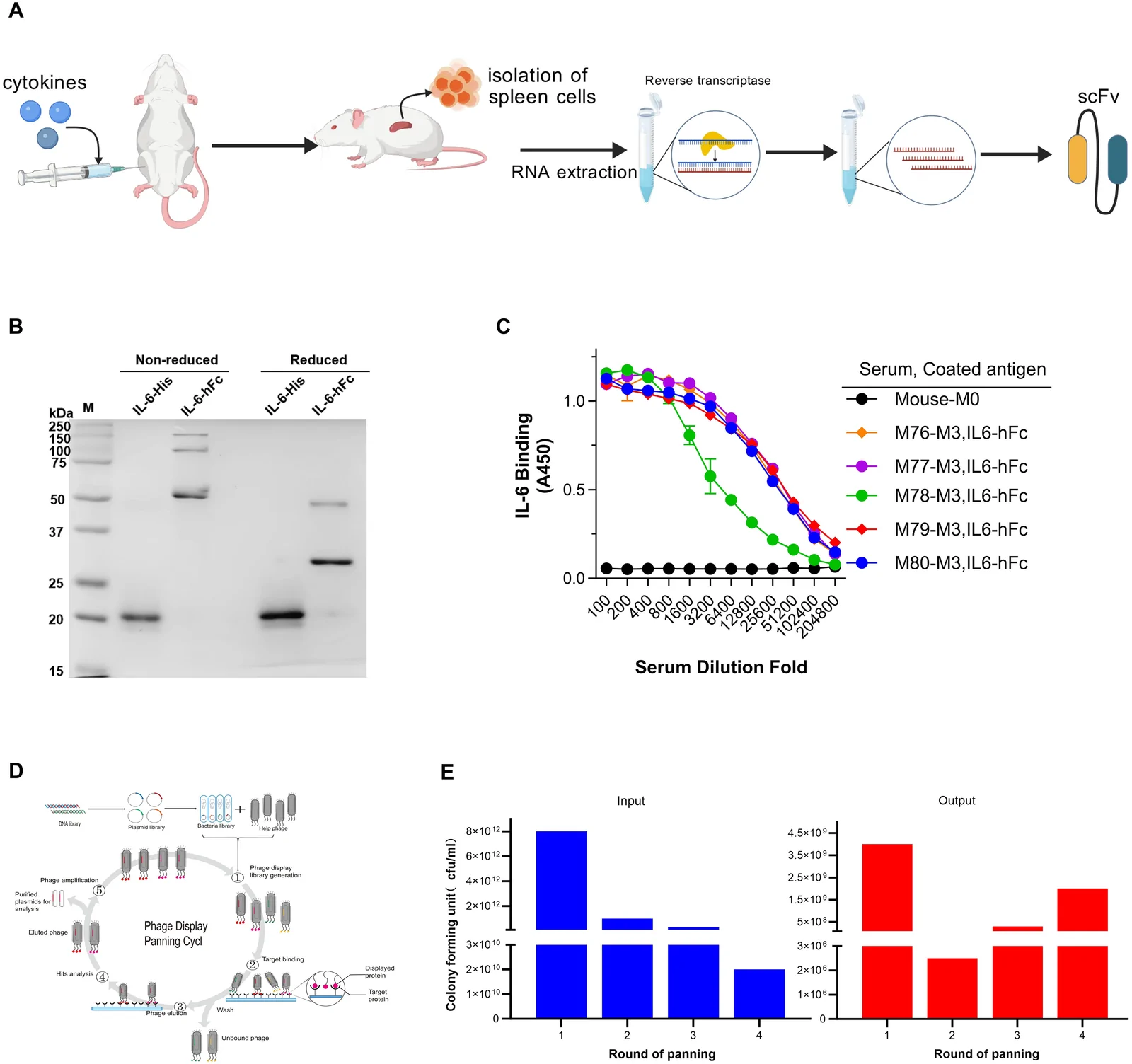
Screening and Validation of Anti-IL-6 Antibodies. **(A)** Antigen immunization and scFv library construction. **(B)** SDS-PAGE of purified IL-6-His/hFc proteins under non-reduced (NR) and reduced (R) conditions. **(C)** Serum titer analysis via ELISA; sera from immunized mice were serially diluted (1:2 from 100×) and detected with HRP-conjugated goat anti-mouse IgG (1:5,000). **(D)** Phage display screening workflow. **(E)** Phage titer quantification during library screening.

BALB/c mice were immunized with IL−6−hFc, eliciting robust antibody titers (**Figure 1A**). Recombinant human IL-6 with a His-tag (IL−6−His, 21.6 kDa) and an IL−6−human Fc (IL−6−hFc, 46.5 kDa) fusion protein was expressed in HEK−293F cells and subsequently purified (**Figure 1B**). IL−6−hFc appeared as three bands under reducing conditions: the predominant monomer (~46.5 kDa), a dimer (~93 kDa) resulting from Fc−mediated association, and a minor degradation product. Then select the mice with the highest serum titers to construct a phage library (**Figure 1C**). Phage display screening of the resulting splenocyte library then yielded five high−affinity anti−IL−6 antibodies (**Figures 1D, E**), designated M03, M07, M19, M30, and M88.

**Figure 2:**
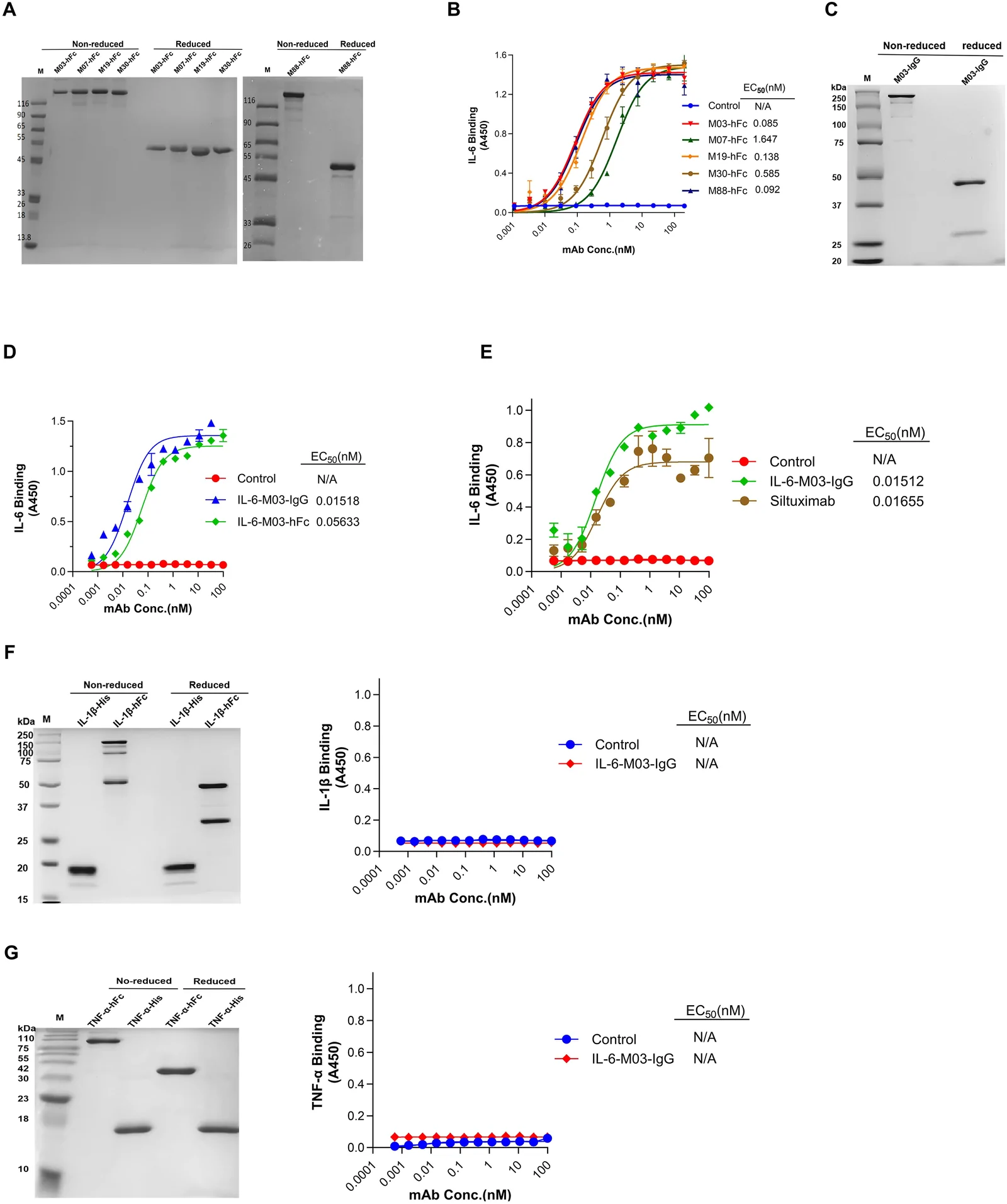
Binding Affinity of Anti-IL-6 Monoclonal Antibodies. **(A)** Reduced (R) and non-reduced (NR) SDS-PAGE of scFv-hFc antibodies (2 µg/lane). **(B)** ELISA-based affinity: IL-6-His-coated plates incubated with serially diluted antibodies (0.01–100 nM), detected with HRP-conjugated goat anti-human IgG (1:5,000). **(C)** NR/R SDS-PAGE of M03-IgG (2 µg/lane). **(D)** Affinity of M03-IgG and M03-hFc (method as in B). **(E)** Affinity of M03-IgG and siltuximab (method as in B). **(F)** SDS-PAGE of purified IL-1β-His/hFc proteins under non-reduced (NR) and reduced (R) conditions. And ELISA-based affinity: IL-1β-His-coated plates incubated with serially diluted antibodies (0.01–100 nM), detected with HRP-conjugated goat anti-human IgG (1:5,000). **(G)** SDS-PAGE of purified TNF-α-His/hFc proteins under non-reduced (NR) and reduced (R) conditions. And ELISA-based affinity: TNF-α-His-coated plates incubated with serially diluted antibodies (0.01–100 nM), detected with HRP-conjugated goat anti-human IgG (1:5,000).

These antibodies were subsequently expressed in CHO cells as scFv−hFc fusion proteins. They achieved yields ranging from 10 to 60 mg/L with purity exceeding 90% (**Figure 2A**). ELISA confirmed that all five clones bound to IL−6−His with nanomolar affinity (**Figure 2B**). Among them, the M03-hFc achieved a yield of 60 mg/L and exhibited the highest binding potency. Subsequently, we converted M03 into a full-length IgG1 format. Under the same CHO cell expression and purification procedure, the final yield of M03-IgG (heavy chain (~50 kDa) or light chain (~25 kDa)) was approximately 150 mg/L, with purity also exceeding 90% (**Figure 2C**). M03-IgG exhibited a higher affinity for IL-6 than M03-hFc, with an EC_50_ of approximately 15 pM (**Figure 2D**).

Siltuximab (brand name Sylvant) is a human−mouse chimeric monoclonal antibody targeting interleukin−6 (IL−6). It functions by blocking the binding of human IL−6 to its receptor, thereby inhibiting IL−6 signaling activity and slowing disease progression. On November 30, 2021, China’s National Medical Products Administration (NMPA) approved the import registration application for injectable siltuximab. This approval was granted through a priority review pathway, designating it as a drug urgently needed for rare diseases. Its approved indication is for the treatment of HIV−negative and human herpesvirus−8 (HHV−8) negative adult patients with idiopathic multicentric Castleman disease (iMCD).

Therefore, we compared the binding affinity of M03−IgG with that of siltuximab. ELISA analysis demonstrated that both antibodies exhibited similarly high affinity for IL−6 (**Figure 2E**). Siltuximab has reported Kd values of ~22 pM (47), whereas M03−IgG exhibits an EC_50_ of ~15 pM, indicates that the affinity of M03-IgG is slightly higher than that of siltuximab. We also expressed and purified two other common inflammatory cytokines, IL-1β and TNF-α, and performed ELISA-based binding assays to demonstrate that IL-6-M03-IgG does not bind nonspecifically to either cytokine (**Figures 2F, G**). This result established M03−IgG as the optimal ligand for subsequent immunoadsorbent development.

### Epoxy microsphere selection and specificity profiling

**Figure 3:**
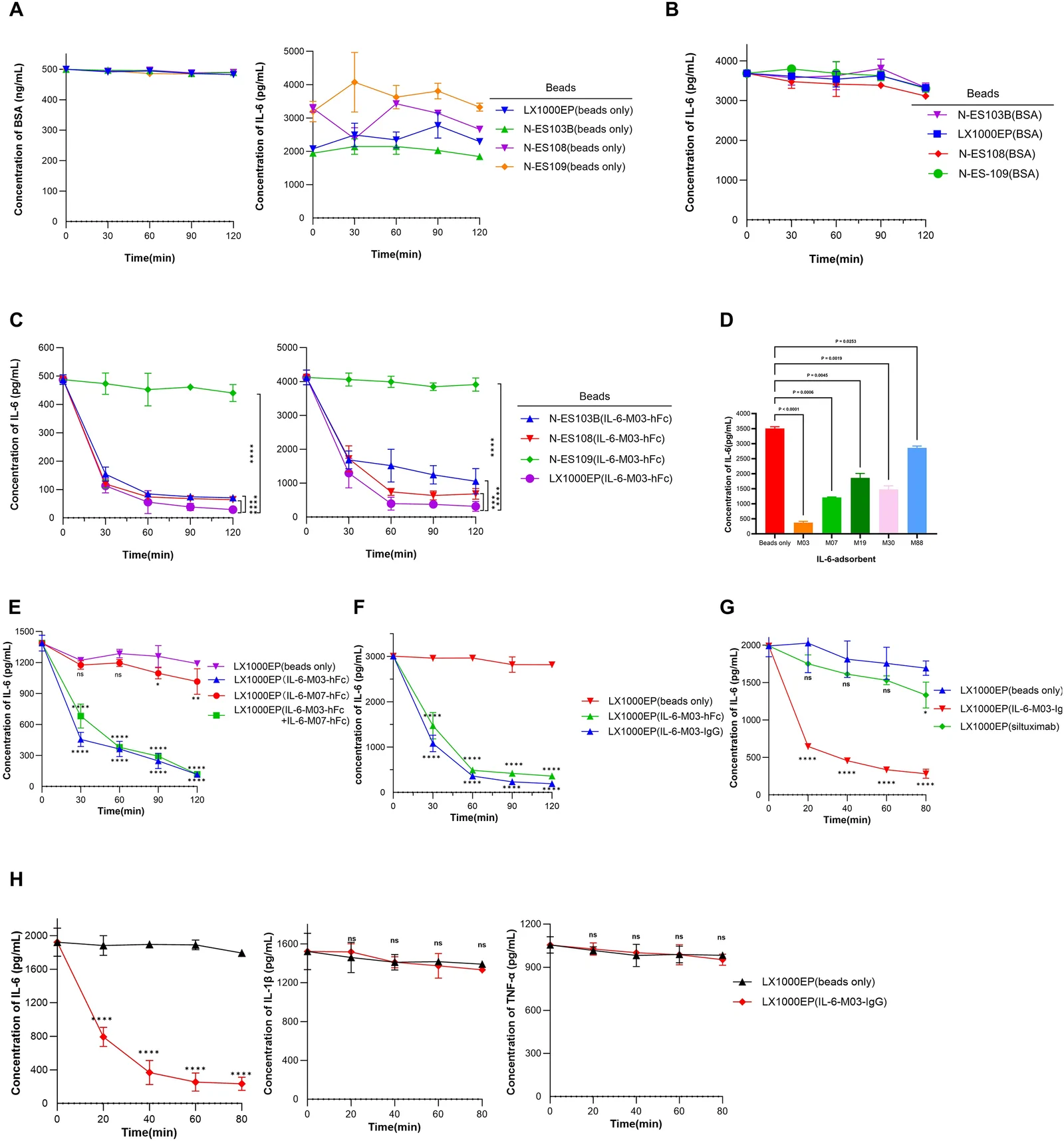
Microsphere Selection and IL-6 Binding Specificity. **(A)** Evaluation of nonspecific adsorption by unloaded microspheres. Using IL-6 and BSA as test analytes, the unloaded microspheres showed no detectable nonspecific physical adsorption. **(B)** Microspheres conjugated with an unrelated protein (BSA), showed no detectable adsorption of IL-6. **(C)** Adsorption of IL-6 by microspheres conjugated with the M03-hFc antibody. Three of the four microsphere types exhibited efficient IL-6 adsorption at both low (500 pg/mL) and high (4000 pg/mL) initial IL-6 concentrations. **(D)** Adsorption efficiency of IL-6 by microspheres conjugated with five different antibodies. The results indicate that microspheres conjugated with the M03 antibody exhibited the highest adsorption efficiency among the five antibodies tested. **(E)** Adsorption efficiency of IL-6 by microspheres conjugated with a mixture of M03 and M07 antibodies. Compared with microspheres conjugated with M03 alone, microspheres conjugated with the M03/M07 antibody mixture did not show improved immunoadsorption efficiency. **(F)** Adsorption efficiency of IL-6 by microspheres conjugated with M03-IgG and M03-hFc constructs. The two constructs exhibited comparable adsorption efficiencies. **(G)** Comparison between microspheres conjugated with siltuximab and those conjugated with M03. Microspheres conjugated with siltuximab showed no detectable adsorption of IL-6. **(H)** Selective specificity of the M03 adsorbent in plasma containing a mixture of cytokines (including IL-6, TNF-α, and IL-1β). Data represent as the mean ± SD(n=3). Statistical analysis for **(C, E-H)** was performed using a two-way ANOVA and performed Tukey’s honestly significant difference test or Dunnett’s test. Statistical analysis for **(D)** was performed using a one-way ANOVA and performed Dunnett’s test. ns, not significant. *p < 0.05, **p < 0.01, ***p < 0.001, ****p < 0.0001. .

Following screening, four types of unloaded epoxy microspheres (LX1000EP, N-ES103B, N-ES108, and N-ES109; particle size, 150–300 μm) showed no nonspecific binding to IL-6 or the BSA control protein in physiological conditions (**Figure 3A**). In addition, BSA-conjugated microspheres did not adsorb IL-6 (**Figure 3B**). These microspheres were therefore selected as carriers for immunoadsorbent preparation.

Three types of microspheres conjugated with the M03-hFc antibody—LX1000EP(M03), N-ES103B(M03), and N-ES108(M03)—specifically adsorbed IL-6 in plasma (**Figure 3C**), among which LX1000EP(M03) exhibited the strongest adsorption efficiency. Among the five antibodies tested, only microspheres conjugated with the M03 antibody exhibited superior immunoadsorption performance (**Figure 3D**), whereas IL-6 adsorption by microspheres conjugated with the other antibodies was relatively weak. When the M07 antibody, which showed the second-highest immunoadsorption performance, was mixed with M03 and co-conjugated onto microspheres, the resulting immunoadsorption performance was comparable to that of microspheres conjugated with M03 alone (**Figure 3E**). This result indicates that incorporation of an additional antibody did not further enhance M03-mediated adsorption. When comparing the two structural formats of the M03 antibody (IgG and hFc), M03-IgG exhibited significantly higher affinity than M03-hFc; however, microspheres conjugated with either format showed comparable IL-6 adsorption performance (**Figure 3F**). These results indicate that both antibody formats are suitable for immunoadsorbent microsphere preparation.

Siltuximab is an FDA-approved monoclonal antibody targeting IL-6. Although its affinity is slightly lower than that of the M03 antibody, siltuximab completely lost its IL-6 binding capacity after conjugation to microspheres (**Figure 3G**). The complete loss of siltuximab binding after immobilization may stem from random orientation, inflexible tethering, or conformational distortion upon surface conjugation, as previously noted for certain antibodies (48). M03−IgG likely possesses a more robust presentation of CDR loops under the same coupling chemistry. These findings further demonstrate that not all antibodies are suitable for immunoadsorbent microsphere preparation, whereas microspheres conjugated with the M03 antibody exhibit superior IL-6 clearance capacity.

To systematically evaluate the targeting specificity of the M03 adsorbent in complex inflammatory environments, as well as its limitations relative to non-selective adsorption techniques, we established an *in vitro* multi-cytokine competition model. In this model, plasma from healthy donors was simultaneously supplemented with three key pro-inflammatory cytokines—IL-6, TNF-α, and IL-1β—to simulate the cytokine profile typically observed during a cytokine storm. Subsequently, adsorption experiments with the M03 adsorbent were performed under dynamic perfusion conditions (flow rate, 5 mL/min; adsorbent-to-plasma ratio, 1:25; duration, 100 min). The results show that the adsorption rates of the M03 adsorbent for the non-target cytokines TNF-α and IL-1β (both average values below 7%) were not significantly different from those of the blank control group, thereby quantitatively confirming its high selectivity (**Figure 3H**; **Supplementary Table 1**). This selective adsorption profile may help preserve essential host immune functions and reduce the risk of immunosuppression associated with broad-spectrum cytokine clearance.

### Immunosorbent preparation and characterization

**Figure 4:**
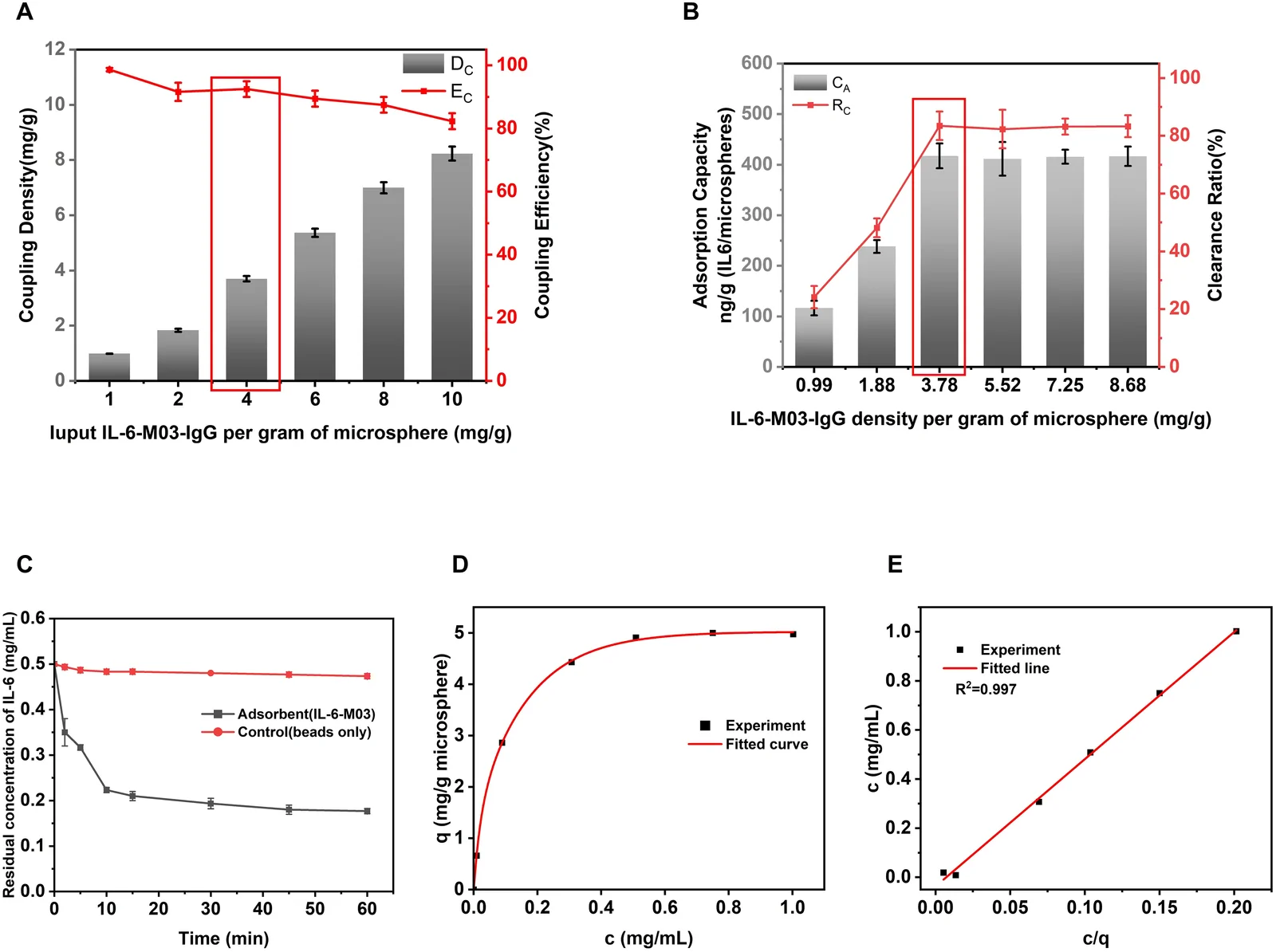
Optimization of IL-6 Antibody Immobilization on Microspheres and Static Adsorption Thermodynamics of IL-6 Immunoadsorbents. **(A)** Coupling density (D_C_, left axis) and efficiency (E_C_, right axis) at varying M03-IgG inputs. Data represent as the mean ± SD (n=3) **(B)** Adsorption capacity (C_A_, left axis) and clearance ratio (R_C_, right axis) of immunosorbents with different D_C_ values. The ratio of microspheres to plasma is 1:25. Data represent as the mean ± SD(n=3). **(C)** Adsorption kinetics: 0.1 g immunosorbent in 0.9 mL IL-6 (0.5 mg/mL). Data represent as the mean ± SD (n=3). **(D)** Langmuir isotherm: binding capacity vs. equilibrium IL-6 concentration. **(E)** Linearized Langmuir isotherm derived from **(D)**. In **(D, E)**, the values of c and q are the average of three independent experiments performed in duplicates.

With 0.5 g of LX1000EP microspheres held constant, the amount of M03-IgG antibody used for conjugation was gradually increased. The results showed that conjugation efficiency (Ec) was highest at an antibody loading of 1 mg/g (antibody/microsphere), approaching 100% (**Figure 4A**). At this loading, the antibody density was 0.99 mg/g of microspheres (**Figure 4B**). With further increases in antibody loading, the coupling efficiency gradually decreased to approximately 80%. When the antibody loading reached 10 mg/g, the coupling efficiency remained above 80%, while the antibody density increased to 8.68 mg/g of microspheres (**Figure 4B**).

Dynamic adsorption experiments were conducted using microspheres with different antibody densities, with the initial IL-6 concentration in plasma set at 20 ng/mL and a microsphere-to-plasma ratio of 1:25 (g/mL). The results showed that when the antibody density (D_C_) exceeded 3.78 mg/g, both the adsorption capacity (C_A_) and removal efficiency (R_C_) approached maximal and saturated levels (**Figure 4B**). Accordingly, an antibody density of 4 mg/g was selected as the optimal coupling density.

### Static adsorption kinetics and isotherm

Equilibrium adsorption was achieved within 30 min (**Figure 4C**). Unmodified microspheres showed no detectable binding to interleukin−6 (IL−6). Langmuir isotherm analysis (R² = 0.997) confirmed a monolayer adsorption mechanism. The analysis yielded a maximum adsorption capacity (q_m_) of 5.18 mg/g and a dissociation constant (K_d_) of 0.036 mg/mL (**Figures 4D, E**; **Supplementary Table 2**).

In solution, the EC_50_ of M03-IgG for IL-6-His was determined by ELISA to be approximately 15 pM (**Figure 2D**), indicating exceptionally high intrinsic binding affinity. In the solid phase immunoadsorption system, static adsorption isotherm data were fitted using the Langmuir model, yielding an apparent K_d_ of 0.036 mg/mL (≈ 1.76 nM). This value represents the free IL-6 concentration required to achieve half-saturation under surface-limited conditions.

### Dynamic perfusion performance

**Figure 5:**
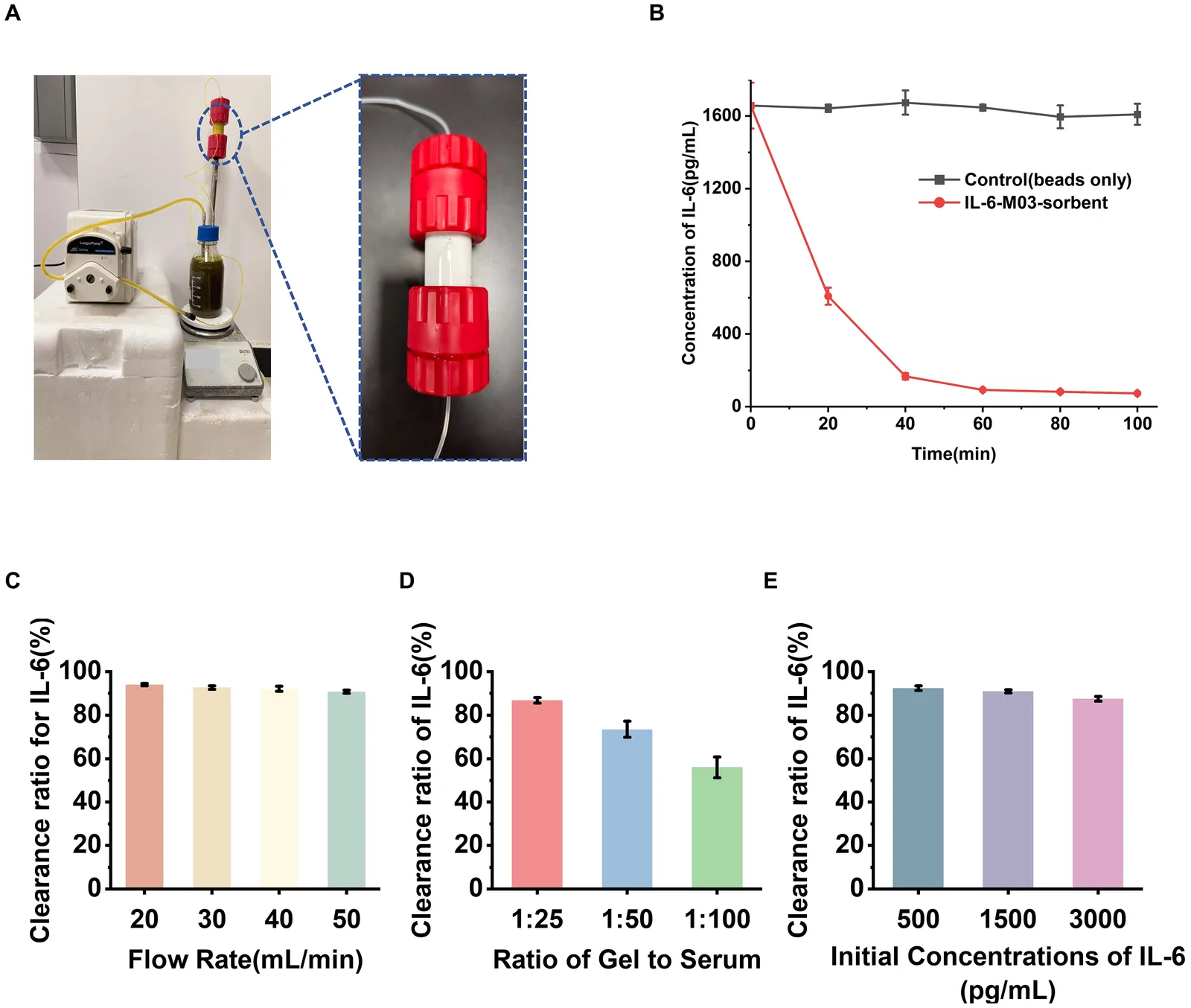
Dynamic IL-6 Adsorption Performance. **(A)** *In vitro* hemoperfusion device. **(B)** Equilibrium time analysis. IL-6 clearance under varying **(C)** flow rates, **(D)** adsorbent-to-serum ratios, and **(E)** initial IL-6 concentrations. Data represent as the mean ± SD(n=3).

When the initial IL-6 concentration in plasma was set to 1600 pg/mL, representative of cytokine storm conditions, IL-6 adsorption reached equilibrium within 100 min in the miniaturized hemoperfusion system (**Figures 5A, B**; **Supplementary Table 3**). The flow rate (20–50 mL/min) had no significant effect on IL-6 clearance, which remained between 90.85% and 94.03% across all tested conditions (**Figure 5C**, **S1A**). Reducing the microsphere-to-plasma ratio from 1:25 to 1:100 resulted in a corresponding decrease in IL-6 clearance from 88.85% to 56.06% (**Figure 5D**, **S1B**). Under optimized conditions (flow rate, 20 mL/min; adsorbent-to-plasma ratio, 1:25), the microspheres consistently achieved an IL-6 clearance of approximately 90% (87.58%–92.44%) across clinically relevant IL-6 concentrations (500–3000 pg/mL) (**Figure 5E**, **S1C**). Detailed performance data for the immunosorbents are provided in **Supplementary Tables 4–6**. Based on the performance parameters (adsorbent-to-plasma ratio 1:25, clearance rate ~90%) obtained from the miniaturized perfusion model established in this study, we performed a theoretical estimation: to process approximately 4 L of patient plasma, a conservative estimate requires about 160 g of antibody-conjugated microspheres. We emphasize that this is only a theoretical projection, and its performance in actual clinical practice will be influenced by many factors such as column design, flow rate, and plasma composition. Future studies need to construct larger-scale pilot devices to verify the feasibility of this estimate.

### Hemocompatibility and stability profiles

**Figure 6:**
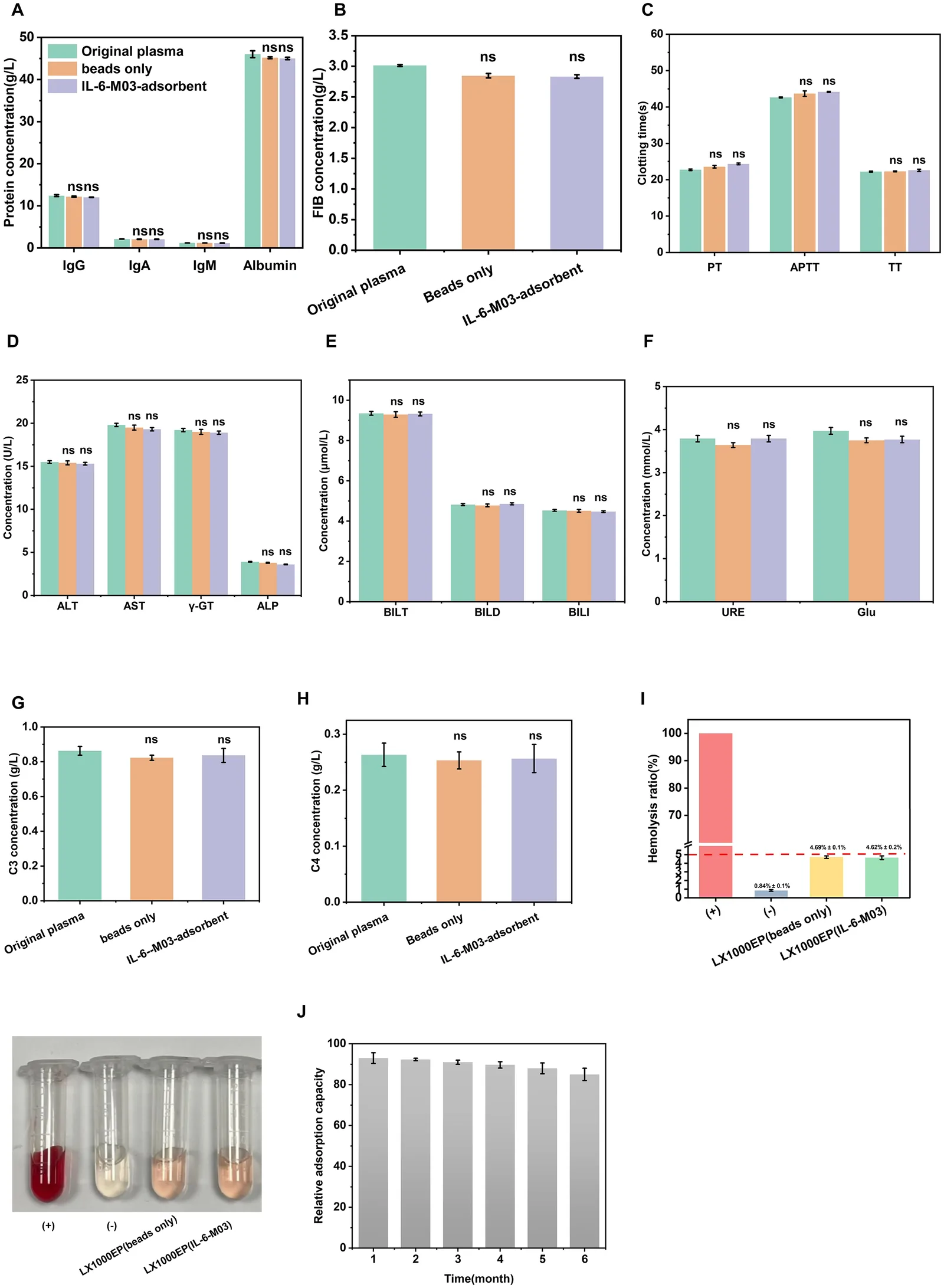
Hemocompatibility and Long-Term Stability. **(A, B)** Plasma protein levels post-adsorption. **(C)** Coagulation times (PT, APTT, TT). **(D)** Key biomarkers of clinical liver damage (ALP, ALT, AST, γ-GT). **(E)** Bilirubin levels (BILT, BILD, BILI). **(F)** Urea (URE) and glucose (Glu) concentrations. **(G, H)** Complement C3/C4 concentrations. **(I)** Hemolysis assay: microsphere-treated RBC suspensions vs. controls. **(J)** Adsorption capacity retention during 6-month storage (4 °C). The values were expressed as a percentage of freshly made M03-adsorbent, which was defined at 100%. All tests were conducted after the immunoadsorbents incubated with human plasma at 37 °C for 60 min. All data represent as the mean ± SD(n=3). Statistical analysis for **(A-H)** was performed using a one-way ANOVA and performed Dunnett’s test. ns, not significant.

The IL−6−targeting immunosorbent demonstrated excellent biocompatibility in comprehensive hemocompatibility assessments. Plasma protein adsorption assays revealed no significant depletion of key proteins, including albumin, immunoglobulins (IgG, IgA, IgM), and fibrinogen—when compared to controls (**Figures 6A, B**), confirming minimal nonspecific binding. Coagulation parameters—prothrombin time (PT), activated partial thromboplastin time (APTT), and thrombin time (TT)—showed no statistically significant differences from the control plasma (**Figure 6C**). The values measured for the immunosorbent (PT: 24.37 ± 0.25 s; APTT: 44.13 ± 0.16 s; TT: 22.60 ± 0.30 s) closely aligned with those of the baseline plasma (PT: 22.73 ± 0.21 s; APTT: 42.63 ± 0.15 s; TT: 22.23 ± 0.15 s). These results indicate that the immunosorbent does not pose a significant risk of inducing either endogenous or exogenous coagulation. Levels of key liver injury markers—alkaline phosphatase (ALP), alanine transaminase (ALT), aspartate transaminase (AST), and γ-glutamyl transferase (γ-GT)—remained unchanged (**Figure 6D**). Plasma bilirubin levels (direct [BILD], indirect [BILI], and total [BILT]) also remained stable, further confirming minimal non-specific binding (**Figure 6E**). Similarly, urea (URE), glucose (Glu) levels were unaffected (**Figure 6F**). Complement activation assays further demonstrated the biocompatibility of the material, as levels of complement components C3 and C4 remained unchanged following plasma contact (**Figures 6G, H**), confirming the absence of complemented inflammatory cascade activation. Hemolysis test results showed that the absorbance of the red blood cell suspension treated with the M03 adsorbent was comparable to that of the negative control group, with a calculated average hemolysis rate of 4.62% ± 0.2%(**Figure 6I**), with hemolysis rates below the 5% safety threshold defined by ASTM F756-2008 (46).

In the evaluation of antibody leaching from immunoadsorbents, a standard curve was plotted by plotting the log_10_-transformed M03-IgG concentration (ng/mL) against the corresponding OD_450_ values, followed by linear fitting (R² > 0.99). The limit of detection was determined to be 0.1 ng/mL(**Supplementary Figure 2**, **Supplementary Table 7**). Results showed that the M03-IgG concentration in all effluent samples was below 0.1 ng/mL, indicating that no detectable antibody leakage occurred under the experimental conditions, confirming the stability of the covalent conjugation between antibody and microsphere.

Long−term storage stability was evaluated in phosphate−buffered saline (PBS; pH 7.4) at 4 °C. The immunosorbent retained 85% of its initial adsorption capacity after six months (**Figure 6J**), validating a robust shelf life suitable for potential clinical deployment.

## Discussion

The development of targeted immunoadsorbents represents a promising therapeutic strategy for managing cytokine-driven pathologies (49). Here, we establish a high-affinity antibody-based platform, designated M03-adsorbent, for the specific removal of interleukin-6 (IL-6), thereby addressing key limitations of current extracorporeal therapies. IL-6 is a pivotal mediator in hyperinflammatory syndromes, and its elevated levels correlate strongly with disease severity in conditions such as COVID-19 (25, 50), sepsis (32), and autoimmune disorders (51–53). Conventional adsorption technologies, exemplified by CytoSorb^®^, rely on nonspecific physicochemical interactions, which result in inconsistent clinical outcomes. As observed in septic or brain-dead patients, these systems achieve only transient IL-6 reduction, with levels rebounding rapidly above baseline within hours (32, 40), and IL-6 clearance rates for CytoSorb^®^ (typically 20–30% over 6 hours) (54); transient reductions followed by rebound phenomena (35); inconsistent mortality benefit in randomized trials (55), indicative of inadequate binding kinetics and capacity. In our dynamic perfusion system with the M03 adsorbent, at a lower adsorbent-to-plasma ratio (1:25), approximately 93% IL-6 clearance was achieved within 100 minutes, and no rebound was observed. Although this is not a direct side-by-side comparison, it clearly indicates that targeted immunoadsorption based on high-affinity antibodies may be superior to adsorption technologies based on non-specific effects in terms of clearance efficiency and kinetics. Compared with non−specific adsorption technologies such as CytoSorb^®^, our M03 adsorbent offers higher specificity. Although the clinical literature used for comparison reports mixed efficacy of CytoSorb^®^, our data indicate that the enhanced target specificity achieved through molecular engineering can translate into more consistent and efficient IL−6 removal, particularly in situations of high antigen burden or when rapid removal of a single key driver is required. We have explicitly added this limitation to the discussion and acknowledge that future studies should include side-by-side experiments. Central to this innovation is M03-IgG, an ultra-high-affinity capture ligand (EC_50_ ≈ 15 pM) isolated through iterative phage display biopanning. M03−IgG displays a binding affinity comparable to that of the clinical antibody siltuximab (**Figure 2e**). Notably, unlike siltuximab, M03−IgG fully retains its binding function after immobilization to microspheres, a critical advantage for immunoadsorbent applications. The observation that siltuximab completely loses IL-6 binding upon immobilization while M03-IgG retains functionality underscores the importance of “solid-phase adaptability” as an intrinsic antibody property. This differential behavior likely arises from a combination of factors related to conjugation chemistry, epitope characteristics, and intrinsic antibody structural properties. First, random epoxy coupling reacts with multiple primary amine groups across the antibody surface, leading to heterogeneous Fab orientations; some Fab fragments may face the microsphere interior or become sterically hindered, reducing antigen accessibility. In contrast, Fc-oriented coupling strategies, such as those using protein A/G or glycan-directed modification, could preserve Fab exposure and maximize binding capacity (56, 57). For siltuximab, preferential coupling at specific Fc residues may further orient the antibody unfavorably compared to M03-IgG, which appears to maintain a more functional orientation. Second, epitope properties may differ: siltuximab may recognize a conformational epitope highly sensitive to spatial distortion upon random multi-point immobilization, whereas the epitope targeted by M03-IgG may be more robust and remain accessible after coupling. Third, the antibody itself differs in conformational flexibility and domain rigidity. Full-length IgG undergoes significant constraints on conformational motion under multi-point covalent attachment, potentially causing CDR distortion or topological hindrance (58). M03-IgG may possess more stable constant domains or more flexible CDR loops that preserve its binding conformation after immobilization (59, 60), whereas siltuximab may lack such structural resilience. These combined factors suggest that “solid-phase fitness” is a multi-dimensional property influenced by both antibody–surface coupling chemistry and the innate structural features of the antibody itself. Future studies could employ molecular dynamics simulations or limited proteolysis coupled with mass spectrometry to validate these structural explanations.

When immobilized onto epoxy-functionalized LX1000EP microspheres at an empirically optimized density of 4 mg/g, the resulting conjugate exhibits favorable binding thermodynamics. Langmuir isotherm analysis confirmed monolayer adsorption, with a maximum capacity (q_m_) of 5.18 mg/g and a dissociation constant (K_d_) of 0.036 mg/mL. We note that the Langmuir apparent Kd obtained in the solid-phase adsorption system (0.036 mg/mL, 1.76 nM) is about 100-fold higher than the EC_50_ of M03-IgG in solution (15 pM). This discrepancy has been qualitatively described in our discussion. We emphasize here that the apparent Kd is a composite parameter reflecting antigen-antibody interactions at the entire solid-liquid interface, which inevitably incorporates non-intrinsic affinity factors such as steric hindrance, mass transfer limitations, surface crowding effects, and microenvironmental changes. Therefore, directly comparing this value with the affinity in solution may be misleading (61). In fact, it is common for reported apparent Kd values of immunoadsorbent systems to be in the nanomolar to micromolar range. We acknowledge that the lack of direct determination of intrinsic affinity and kinetic parameters of M03-IgG using label-free techniques such as surface plasmon resonance (SPR)/bio-layer interferometry (BLI) is a limitation of this study. Future work will be dedicated to determining its precise Kd value by SPR and further evaluating the components of the apparent Kd using techniques such as quartz crystal microbalance with dissipation monitoring (QCM-D). These metrics reflect efficient binding site utilization without steric interference, a known limitation in antibody-functionalized systems (62). Importantly, this molecular precision prevents the nonspecific adsorption of essential plasma proteins, a major drawback of polymeric adsorbents that deplete albumin, immunoglobulins, and coagulation factors.

The clinical translatability of this platform is supported by its robust performance under simulated hemoperfusion conditions. Its insensitivity to physiological flow rates (20–50 mL/min) indicates adaptability to variable patient hemodynamics. Furthermore, the 1:25 adsorbent-to-plasma ratio provides a scalable treatment paradigm (e.g., 160 g adsorbent per 4 L plasma volume) that is compatible with existing perfusion circuits. Comprehensive biocompatibility assessments further substantiate their clinical potential. The system demonstrated negligible effects on coagulation parameters (PT, APTT, TT), complement activation (stable C3/C4 levels), and essential protein depletion, meeting critical safety thresholds for blood-contacting biomaterials. It is worth noting that the average hemolysis rate we measured was 4.2%, which is close to the safety upper limit of 5%. This finding suggests that, to avoid potential hemolysis risks, the M03 adsorbent may be more suitable for plasma adsorption (plasmapheresis) mode rather than direct whole blood perfusion in the early stages of clinical application. Integrating the adsorbent into a standard plasma separation circuit is a mature and safe technique that completely avoids direct contact between red blood cells and the adsorbent. Future work can explore further hydrophilic modification of the microsphere surface to reduce its hemolysis rate in whole blood, thereby expanding its application scenarios. Long-term stability data, showing 85% capacity retention after 6 months at 4 °C, further support deployment readiness by addressing a key requirement for clinical adsorbents, where shelf life directly impacts therapeutic accessibility.

Our findings should be considered within the context of emerging therapeutic paradigms. Although IL-6 is a master regulator of inflammatory cascades, clinical evidence indicates incomplete responses to monotherapy in conditions such as rheumatoid arthritis and Still’s disease (20), highlighting the potential benefits of multi-cytokine targeting. The modular design of our platform allows for the future integration of antibodies targeting co-pathogenic mediators such as TNF-α, IL-1β, IL-8, IL-17A, or GM-CSF. Recent advances in biparatopic nanobodies and bispecific antibody engineering (9, 63–65) could further enable the development of next-generation adsorbents with broader cytokine capture spectra.

The significance of this study extends beyond the development of an effective IL-6 scavenger to the establishment of a workflow for evaluating solid-phase fitness and the identification of M03-IgG as a promising scaffold antibody for such applications. Defined by high affinity, tolerance to immobilization, low nonspecific adsorption, and long-term storage stability, M03-IgG may provide a useful template for the development of future immunoadsorbents. More broadly, successful expansion to multi-target removal will likely require not the simple combination of conventional antibodies, but the selection of ligands that retain function after co-immobilization. Otherwise, the performance of bispecific or multivalent constructs may remain constrained by the inactivation of individual components. Accordingly, this work offers not only a candidate adsorbent, but also a general design principle for scalable immunoadsorption technologies: ligand performance under solid-phase conditions should be established before system-level integration.

Several limitations of this study should be acknowledged. First, all findings presented herein are derived from *in vitro* experiments. Although our dynamic perfusion model was designed to simulate clinically relevant conditions, it cannot fully recapitulate the complex hemodynamic environment, immune system feedback, and inter-organ interactions present *in vivo*. Therefore, the actual therapeutic efficacy, safety profile, and biodistribution of the M03 adsorbent require validation in appropriate animal models, such as the cecal ligation and puncture (CLP)-induced sepsis model or the lipopolysaccharide (LPS)-induced cytokine storm model. Future work will focus on evaluating the therapeutic efficacy and biosafety of this platform in both small and large animal models to support translational advancement.

Second, the specificity assessment of the M03 adsorbent was limited to three major pro-inflammatory cytokines (IL-6, TNF-α, and IL-1β). We acknowledge that clinical cytokine storm syndromes involve a broader spectrum of soluble mediators, including IL-8, IL-10, IFN-γ, and GM-CSF. Although M03-IgG exhibits no specific binding to these factors, the potential for non-specific adsorption and the possible interference by high-abundance plasma proteins under more complex inflammatory milieus remain to be systematically investigated. Future evaluations should incorporate a wider panel of inflammatory mediators to comprehensively delineate the selectivity profile of this platform.

Third, the regeneration and reusability of the M03 adsorbent were not assessed in this study. Given the irreversible nature of covalent antibody immobilization, developing mild elution conditions (e.g., low-pH buffers or chaotropic agents) that preserve antibody integrity without denaturation represents a critical technical challenge for IgG-based systems. Efficient and sustainable regeneration strategies are essential for reducing treatment costs and enhancing clinical practicality. Accordingly, future research should prioritize the optimization of regeneration protocols and the evaluation of adsorption capacity retention over multiple adsorption–elution cycles.

Despite these limitations, our study establishes a foundational framework for high-performance immunoadsorbent design. The M03-IgG antibody demonstrates exceptional “solid-phase fitness,” and the platform offers a modular architecture amenable to future integration with multi-cytokine targeting strategies. Addressing the above limitations will be essential to advance this technology toward clinical translation.

## Conclusions

This study reports on the development of a high-performance immunoadsorbent designed for the targeted removal of interleukin-6 (IL-6). The platform is based on the M03-IgG antibody, an ultra-high-affinity capture ligand (EC_50_ ≈ 15 pM) identified through phage display screening, which was subsequently immobilized onto epoxy-functionalized LX1000EP microspheres. The resulting M03-adsorbent exhibits high specificity and capacity, achieving 93% IL-6 clearance within 100 minutes in a dynamic perfusion model under clinically relevant conditions (adsorbent-to-plasma ratio of 1:25). Adsorption characteristics were assessed via Langmuir isotherm analysis, which indicated monolayer adsorption with a maximum capacity (q_m_) of 5.18 mg/g and a dissociation constant (K_d_) of 0.036 mg/mL. Biocompatibility evaluations demonstrated minimal nonspecific protein adsorption, negligible effects on coagulation parameters (PT, APTT, TT), and no significant complement activation. The adsorbent also retained 85% of its initial binding capacity after six months of storage at 4 °C, indicating favorable long-term stability. M03-IgG represents a rare example of a therapeutically relevant antibody with inherent solid-phase fitness. Rather than merely enabling IL-6 removal, it provides a validated, transferable scaffold for engineering high-fidelity immunoadsorbents—shifting the field’s focus from “target selection” to “ligand engineering.” This work establishes a functional benchmark and a practical framework for developing next-generation, antibody-based blood purification technologies. Together, these results establish a robust and adaptable platform for the extracorporeal treatment of IL-6-driven hyperinflammatory syndromes, with a modular design that permits future integration of antibodies against other pathogenic cytokines.

## Data Availability

The original contributions presented in the study are included in the article/**Supplementary Material**, further inquiries can be directed to the corresponding author/s.
