# Correction: A robust and scalable immunoadsorbent platform based on the high-affinity M03-IgG antibody for targeted IL-6 Removal in cytokine storm

**DOI:** 10.3389/fimmu.2026.1961570

**Published:** 2026-08-17

**Authors:** Yaxi Yang, Lei Wan, Youjia Qin, Jiangtao Guo, Yong Wang, Kangkang Ji, Sheng Hu, Mingqian Feng

**Affiliations:** 1 College of Biomedicine and Health, Huazhong Agricultural University, Wuhan, Hubei, China; 2 College of Life Science and Technology, Huazhong Agricultural University, Wuhan, Hubei, China; 3 Department of Respiratory and Critical Care Medicine, Binhai County People’s Hospital, Binhai Clinical College, Yangzhou University Medical College, Yancheng, Jiangsu, China; 4 Department of Medical Oncology, Tongji Medical College, Hubei Cancer Hospital, Huazhong University of Science and Technology, Wuhan, Hubei, China

**Keywords:** cytokine removal, hypercytokinemia, il-6, immunoadsorption, inflammation

Affiliation “Department of Respiratory and Critical Care Medicine, Binhai County People’s Hospital, Binhai Clinical College, Yangzhou University Medical College, Yancheng, Jiangsu, China” was omitted from the paper. This affiliation has now been added for author(s) “Kangkang Ji”.

Affiliation “Department of Oncology, Hubei Cancer Hospital, Tongji Medical College, Huazhong University of Science and Technology, Wuhan, Hubei, China” was omitted from the paper. This affiliation has now been added for author(s) “Sheng Hu”.

The original version of this article has been updated.

