# Supplementary material for: A robust and scalable immunoadsorbent platform based on the high-affinity M03-IgG antibody for targeted IL-6 Removal in cytokine storm
Source: Front Immunol. 2026 Jul 24;17:1907563. doi: 10.3389/fimmu.2026.1907563 (PMC13449301; doi:10.3389/fimmu.2026.1907563)
Supplement: Supplementary file 1 [file Supplementaryfile1.zip › supplementary material/Supplementary figure legend.docx]

**Figure S1. Comparison of IL-6 adsorption in the in vitro dynamic adsorption model.**
Equilibration time of immunosorbents in the dynamic adsorption test. Removal effects on IL-6 under different (A) flow rates, (B) sorbent-to-serum ratios, and (C) initial IL-6 concentrations.In (A−C), the data are presented as means ± standard deviation, n = 3.
**Figure S2. Standard curve for the M03-IgG ELISA.**

Recombinant M03-IgG at known concentrations was used as standards in a serial dilution series. The x-axis represents the logarithm (base 10) of the M03-IgG concentration (ng/mL), and the y-axis represents the absorbance (OD) at a wavelength of 450 nm. Data are presented as mean ± standard deviation(n=3).
