# Supplementary material for: A robust and scalable immunoadsorbent platform based on the high-affinity M03-IgG antibody for targeted IL-6 Removal in cytokine storm
Source: Front Immunol. 2026 Jul 24;17:1907563. doi: 10.3389/fimmu.2026.1907563 (PMC13449301; doi:10.3389/fimmu.2026.1907563)
Supplement: Supplementary file 1 [file Supplementaryfile1.zip › supplementary material/Supplementary Table.docx]

**Table S1**. Clearance ratio of IL-6 by immunadsorbents in a multicytokinic competition assay. The initial concentrations of IL-1β, IL-6, and TNF-α were 1500, 1900, and 1000 pg/mL, respectively.

| Immunosorbent | M03-sorbent | | |
| --- | --- | --- | --- |
| Equilibrium concentration(pg/mL)  Clearance ratio (%) | IL-1β | IL-6 | TNF-α |
|  | 1409.7±15.3 | 232.9±31.8 | 953.0±15.8 |
|  | 6.01±1.0 | 87.7±1.6 | 4.7±1.6 |

**Table S2**. Initial and residual concentrations of IL-6 in static adsorption for M03-sorbent.

| Initial concentrations (mg/mL) | Residual concentrations (mg/mL) |
| --- | --- |
| 0 | 0 |
| 0.075 | 0.00893±0.000416 |
| 0.375 | 0.0189±0.000351 |
| 0.75 | 0.307±0.00753 |
| 1 | 0.509±0.00149 |
| 1.25 | 0.75±0.00872 |
| 1.5 | 1.0024±0.0141 |

**Table S3**. Residual concentrations of IL-6 with time. Initial concentrations of IL-6 was 1600 pg/mL and ratio of gel to serum was 1:25.

| Time (min) | Residual concentrations of IL-6 (pg/mL) | |
| --- | --- | --- |
|  | M03-sorbent | Control（Beads only） |
| 0 | 1676.071±109.11 | 1657.61±125.75 |
| 20 | 583.92857±61.53 | 1643.33±20.27 |
| 40 | 368.92±77.68 | 1674.46±66.71 |
| 60 | 192.64±55.49 | 1647.04±16.75 |
| 80 | 150.78±20.32 | 1596.4±63.22 |
| 100 | 150.71±13.06 | 1610±57.99 |

**Table S4**. Clearanceratio of immunosorbents for different flow rate. Initial concentrations of IL-6 was 4500 pg/mL, and ratio of gel to serum was 1:25.

| Immunosorbent | Clearance ratio (%) | | | |
| --- | --- | --- | --- | --- |
|  | 20(mL/min) | 30(mL/min) | 40(mL/min) | 50(mL/min) |
| M03-sorbent | 94.03±0.53 | 92.75±0.8 | 92.12±1.23 | 90.84±0.73 |

The volume of circulating serum: 500 mL. Circulation time: 100 min.

**Table S5**. Clearance ratio of immunosorbents for different ratio of gel to serum. Initial

concentrations of IL-6 was 1400 pg/mL, and flow rate was 20 mL/min.

| Immunosorbent | Clearance ratio (%) | | |
| --- | --- | --- | --- |
|  | 1:25 | 1:50 | 1:100 |
| M03-sorbent | 86.84±1.23 | 73.54±3.7 | 56.06±4.84 |

The volume of circulating serum: 500 mL. Circulation time: 100 min.

**Table S6**. Clearance ratio of immunosorbents for different initial concentrations of IL-6. Ratio of gel to serum was 1:25 and flow rate was 20 mL/min.

| Immunosorbent | Clearance ratio (%) | | |
| --- | --- | --- | --- |
|  | 500pg/mL | 1500pg/mL | 3000pg/mL |
| M03-sorbent | 92.44±1.07 | 90.96±0.78 | 87.57±1.03 |

The volume of circulating serum: 500 mL. Circulation time: 100 min.

**Table S7**. ELISA quantification of M03-IgG in effluent samples from dynamic perfusion experiments.

| Sample number | Mean OD₄₅₀ ± SD (n=3) | Calculated Concentration (ng/mL) | Final Concentration (ng/mL) |
| --- | --- | --- | --- |
| Effluent 1 | 0.0818± 0.004 | 2.82181 | < 0.1 |
| Effluent 2 | 0.0873± 0.005 | 2.85756 | < 0.1 |
| Effluent 3 | 0.0833± 0.009 | 2.83158 | < 0.1 |
| Effluent 4 | 0.0920± 0.006 | 2.88909 | < 0.1 |
| Effluent 5 | 0.0873± 0.005 | 2.85756 | < 0.1 |
| Control plasma | 0.0854± 0.003 | 2.84508 | < 0.1 |
| Blank (PBS) | 0.0642± 0.005 | — | — |
